# Watery diarrhea in early childhood and neuro-cognitive development: longitudinal follow-up study in Bangladesh

**DOI:** 10.3389/fpubh.2026.1835082

**Published:** 2026-08-18

**Authors:** Bharati Rani Roy, Mohammad Ali, Mahbub Elahi, Dilara Begum, Sampa Dash, Eva Sultana, Kaniz Fatima, Shamim Al Mamun, Farina Naz, Malathi Ram, Tahmeed Ahmed, A. S. G. Faruque, Subhra Chakraborty, Fahmida Tofail

**Affiliations:** 1 International Centre for Diarrheal Disease Research, Mohakhali, Dhaka, Bangladesh; 2 Bloomberg School of Public Health, Johns Hopkins University, Baltimore, MD, United States

**Keywords:** Bangladesh, diarrhea, early childhood, neuro-cognitive, watery

## Abstract

**Introduction:**

Globally, diarrheal disease is the third leading cause of under-five child mortality. Early-life diarrhea may adversely affect neurodevelopment through nutrient loss, environmental enteropathy, and inflammation; however, longitudinal evidence remains limited. We investigate the relationship between watery diarrhea and neurocognitive development among children under 2 years old in rural Mirzapur, Bangladesh.

**Methods:**

In this prospective longitudinal study, 138 children aged <24 months presenting with WD were enrolled. Neurodevelopment was assessed using the Bayley Scales of Infant and Toddler Development-4 at 2 days post-recovery and at 90 days follow-up. Associations between developmental scores (cognitive, language, motor) and diarrheal duration, severity, hospitalization, anthropometry, hygiene practices, home stimulation, and socioeconomic factors were examined using multivariable linear regression models.

**Results:**

The mean age of participants was 10.8 ± 5.9 months, and 60.9% were male. Median diarrheal duration was 176 h (IQR: 120–238). At baseline, mean (±SD) cognitive, language, and motor scores were 97.3 ± 11.0, 97.9 ± 11.5, and 103.8 ± 11.5, respectively. In adjusted analyses, boys consistently demonstrated significantly higher language scores than girls. Children’s age and greater home stimulation were significantly associated with higher cognitive and motor scores soon after recovery, with sustained effects for cognition and language at 90 days. Diarrheal duration >72 h and lower height-for-age z-scores in Pediatrics were significantly associated with poorer language development, while hospitalization was associated with reduced motor scores shortly after recovery; however, these effects improved by 90 days. Improved handwashing practices were associated with higher language and motor scores at follow-up.

**Conclusion:**

Watery diarrhea in early childhood was associated with short-term impairments in neurodevelopment, particularly in language and motor domains, with partial recovery by 90 days. Child sex and age, diarrheal duration, hospitalization, caregiving environment, and hygiene practices influenced recovery. Integrated interventions addressing both diarrhea and the post-diarrheal caregiving environment are essential to support early child development.

## Introduction

According to the World Health Organization (WHO), diarrheal disease is defined as the passage of three or more loose or liquid stools per day, or more frequent passage than is normal for the individual (1, 2). It remains a leading cause of morbidity and mortality among children under five, particularly in low- and middle-income countries (LMICs), accounting for nearly 9% of deaths and affecting 1.7 billion children annually (1, 3, 4). In Bangladesh, the prevalence of diarrhea among children under 5 years of age has been reported to be 17% (5). However, its sequels in growth and development, particularly in early years during the critical period of brain development, are less explored. Diarrhea can be categorized into acute, persistent, and chronic based on its duration (1). Diarrhea lasting 14 days or longer, categorized as persistent, has long-term consequences on cognitive development (6). Beyond its acute health consequences, early-life diarrheal illnesses, particularly when frequent or persistent, may have long-term impacts on child growth and neuro-cognitive development (6). Neurocognitive development refers to the development of brain-related functions, particularly cognitive abilities such as thinking, learning, memory, language, and problem-solving, as part of a child’s overall development (7). In a Bangladeshi cohort, Norovirus, Sapovirus, and typical Enteropathogenic *Escherichia coli* (EPEC) were identified as significant enteric pathogens associated with childhood growth faltering and neurodevelopment (8). Recurrent enteric infections during the first 2 years of life can impair nutrient absorption, while recurrent diarrheal illness may disrupt the intestinal barrier and contribute to chronic inflammation, factors that collectively have the potential to adversely affect neurodevelopment in young children (9, 10). Therefore, diarrhea may contribute to cognitive impairment through the well-documented nutrition-infection cycle (11). An interventional study found that diarrhea, either alone or along with growth (weight)-related nutritional deficiencies, may impair cognitive development (12). Additionally, early childhood stunting, a common outcome of the nutrition-infection cycle, has been linked to cognitive impairment (13, 14). Moreover, studies suggested that an imbalance in the gut microbiota, often triggered by recurrent diarrheal episodes, may contribute to gut-brain axis dysfunction, ultimately leading to impaired cognitive development (15). Diarrheal disease continues to be a significant cause of inflammation in pediatric populations in LMICs, and several studies have directly linked it to poor neurodevelopment in these children (6, 11, 16). Febrile illness and systemic inflammatory cytokines have been linked to lower neurodevelopmental scores in children from LMICs, adding further evidence of the biological mechanisms that connect infection and brain function (17). The impact of chronic systemic inflammation on child cognitive development is increasingly recognized as a major factor impairing millions of children worldwide from reaching their full developmental potential (18, 19).

Several large-scale studies have investigated that early childhood diarrhea was negatively associated with later cognitive development and school performance (6, 16, 20). The Malnutrition and Enteric Disease (MAL-ED) study found that diarrheal pathogens, such as sapovirus and enteropathogenic *Escherichia coli* (EPAC) have been negatively associated with cognitive development, while norovirus GII was positively associated with cognitive development and growth (8). A pooled analysis of four longitudinal cohorts found that diarrhea prevalence in the first 2 years of life was not independently associated with later cognitive outcomes, whereas length-for-age Z-scores were consistently predictive across sites (21). These findings suggest that linear growth may mediate the relationship between early infections and neurodevelopment, but the independent role of diarrhea remains uncertain.

Despite being a major threat to child health and neurocognitive development, diarrhea remains an under-researched area in terms of its long-term developmental impact.

Previous studies have highlighted the need to explore the complex interactions between infections such as diarrhea, nutrient absorption, malnutrition, and impaired cognitive development (22). The present study, therefore, investigates the relationship between watery diarrhea and neurocognitive development among children under 2 years old in rural Mirzapur, Bangladesh.

## Materials and methods

### Study site and participants

This study was carried out in Mirzapur, a rural subdistrict of Tangail district, Bangladesh, located approximately 60 km northwest of Dhaka. The study area is covered by the Mirzapur Health and Demographic Surveillance System (HDSS) of icddr,b, established in 2007, which conducts regular surveillance of approximately 300,000 individuals from 77,000 households. Within the HDSS area, participants were recruited from Kumudini Hospital, a 750-bed nonprofit hospital established in 1938 that provides healthcare services to surrounding rural communities. The hospital operates a dedicated diarrhea treatment unit managing approximately 1,500 diarrheal cases annually and maintains strong community engagement through outreach activities and subsidized care, which facilitates participant retention in research studies. Previous surveillance data from Mirzapur demonstrated a high burden of Shigella infection among children aged 12–59 months presenting with diarrhea.

A total of 138 children aged >1 to <24 months with acute watery diarrhea were enrolled between November 8, 2021, and October 2, 2022, as part of the ongoing Impact of Non-Dysenteric Shigella Infection on the Growth and Health of Children over Time (INSIGHT) study (23).

### Inclusion and exclusion criteria

The study included children aged >1 month to <24 months who sought care for watery diarrhea at Kumudini Hospital, resided within the hospital catchment area, and were available for sample and data collection during scheduled follow-up visits. Children were excluded if diarrhea onset had occurred more than 48 h before enrollment, if they had received antibiotics for diarrhea within the preceding 3 days or were prescribed antibiotics for the current episode, or if they had a concurrent severe illness requiring specific treatment. Additional exclusion criteria included severe acute malnutrition (weight-for-length z-score < −3) or the presence of bilateral edema.

### Sampling technique

All children presenting to Kumudini Hospital with diarrhea initially received the standard of care from hospital physicians in accordance with the national treatment guidelines and hospital protocols of Bangladesh. Standard management included rehydration therapy and zinc supplementation for all children with diarrhea, depending on the degree of dehydration. Participants were selected using a convenience sampling technique. During the study period, children aged 1–24 months who were admitted to the hospital with diarrheal illness were assessed for eligibility according to the predefined inclusion and exclusion criteria. Written informed consent was obtained from their parents before participation in the study.

### Measurement tools for mothers

*HOME*, we used the Home Observation for Measurement of the Environment (HOME) after resolving the index diarrhea episode. This is a widely used inventory and adapted tool for Bangladeshi children (24). This tool has been widely used and adapted for use among Bangladeshi children. It was administered through household visits by trained field staff. Caregivers were interviewed, and the home environment was directly observed to assess parent–child interactions, environmental safety, cleanliness, and opportunities for play and learning (25, 26). The HOME inventory is a widely used standardized tool designed to assess the quality and quantity of stimulation and support available to a child in the home environment. It evaluates multiple dimensions, including caregiver-child interaction, availability of learning materials, and the overall emotional and cognitive stimulation within the household. In this study, information was collected from the child’s primary caregiver through structured interviews conducted during household visits by trained field staff. In Bangladesh, the HOME inventory has been adapted and applied in several child development and public health studies to ensure cultural relevance and contextual appropriateness, particularly in rural and low-resource settings. The adapted version retains the core constructs of the original instrument while incorporating locally relevant caregiving practices and play materials. This version has been widely used and validated in studies conducted by icddr,b and other child development research in Bangladesh.

*Socio-demographic questionnaire*, Household socioeconomic status was measured using information on parental education, occupation, income, household assets, housing characteristics, along with household hygiene practices during cooking and child feeding through a structured questionnaire. We obtained this information form mother of the finally enrolled children during the baseline interview.

*Morbidity Questionnaire,* Hospitalization history, and diarrheal morbidities such as duration of diarrhea, maximum number of loose stools per day, duration of vomiting in days, confirmed temperature, and dehydration data were collected through the morbidity questionnaire. This information was collected from the mothers of the enrolled children through one-to-one interviews, except for hospitalization history at baseline. In addition, the hospitalization history of the participants was collected from day three onward.

### Tools for children

*Bayley-4,* Children’s neurocognitive development was assessed using the Bayley Scales of Infant and Toddler Development, fourth edition (Bayley-4). The Bayley-4 is a standardized instrument used to evaluate the cognitive, language, motor, and socioemotional development of infants and toddlers aged 1–42 months (27). The cognitive domain comprised 81 items. The language domain included receptive (42 items) and expressive (37 items) subdomains, while the motor domain included fine motor (46 items) and gross motor (58 items) subdomains. Each assessment took approximately 50–60 min and was conducted in a quiet, well-lit, and distraction-free field office setting by the same tester in a single session, with the child seated alongside the mother. Two female Field Research Assistants received 1 month of intensive training from a senior psychologist on rapport building and standardized, nonjudgmental test administration. Data collection commenced only after achieving an inter-rater agreement of over 85%. Children were assessed in a clinic setting in a well-ventilated, well-lit room with minimal distractions. Caregivers were present during the assessment; however, they were instructed not to provide direct assistance or prompt the child during testing.

*Anthropometry*, Child’s weight, length, mid-upper arm circumference, and head circumference were measured at the baseline, followed by days 14, 30, 60, and 90. In this analysis, we used baseline and 90-day follow-up data.

### Data collection

If the caregiver expresses interest, the study staff or clinician will provide the study information sheet/flyer. Caregivers of children meeting the preliminary eligibility criteria and expressing continued interest will undergo the pre-screening consent process. Eligible participants will subsequently proceed to the full informed consent process. The study clinician will be responsible for assessing eligibility criteria and documenting clinical symptoms according to the study protocol. The clinician will also receive training on the informed consent procedures. Neurocognitive parameters were measured first at 2–7 days after recovery from the index diarrhea to avoid any impact of the agitation from the diarrhea episode on neurocognitive measurements. The second measurement was taken at 90 days after enrollment.

### Operational definition

*Diarrheal duration*, we calculated diarrheal duration in hours by subtracting the index diarrhea resolve date from the index diarrhea onset date. A day of diarrhea was defined as one in which a child passed three or more liquid or loose stools (taking the shape of the container) during 24 h.

*Diarrheal episodes* were defined as at least 1 day of diarrhea followed by 3 or more diarrhea-free days.

### Statistical analysis

In this study, descriptive statistics were used to summarize the socio-demographic characteristics and mean value of nutritional status, exposure, and outcome variables. Continuous variables were presented as means and standard deviations, while categorical variables were presented as frequencies and percentages. The crowding index was calculated by dividing the number of household members by the number of rooms. Monthly household income was categorized into three groups based on the distribution of the study data. The median income (BDT 20,000) was used as the central cut-off to define lower and higher income groups, with an additional middle category created to preserve distributional balance for analysis. For developmental assessment, a polytomous scoring system was applied, assigning scores of 2 for mastery-level responses, 1 for emergent responses, and 0 for no response. Raw scores were subsequently converted into age-adjusted scaled scores and standardized composite scores following the Bayley-4 manual (mean = 100, SD = 15). For bivariate analyses, we assessed the associations between developmental outcomes (cognitive, language, and motor) and the independent variables (child’s sex, crowding index, hygiene, diarrheal duration, diarrheal severity, and fever), using independent sample t-tests. Spearman’s rank correlation coefficients were calculated to evaluate the relationships between developmental outcomes and the child’s age, caregiver education, household income, nutritional status, and home stimulation. For multivariable analysis, multiple linear regression models were used to identify the associations of cognitive, language & motor with diarrheal duration adjusted for child age, sex, nutritional status, caregiver education, crowding index, household income, maintaining hygiene during cooking and feeding children, temperature, and total home. Adjusted beta coefficients and their 95% confidence intervals (CIs) were reported. Statistical significance was set at *p* < 0.05 for all analyses. All statistical tests were two-tailed.

HOME Inventory items were scored according to standard guidelines and summed to generate a total HOME score, with higher scores indicating a more stimulating and supportive home environment. The total HOME score was used as a continuous variable and included in bivariate and multivariable analyses to examine its association with child developmental outcomes.

We evaluated the diarrheal severity by the modified Vesikary Score System (VSS); a total score <5 indicates mild diarrhea, and a score of 6 to 9 indicates moderate to severe diarrhea (28). The modified VSS has a 0-15-point numerical score, including the assessment for the presence of several clinical outcomes, such as duration of diarrhea, maximum number of loose stools per day, duration of vomiting in days, confirmed temperature, and dehydration. We used the Statistical Package for the Social Sciences 20 version (SPSS 20).

## Results

### Study participants characteristics

The characteristics of the study population are shown in Table 1. The mean (SD) age of the children was 10.84 ± 5.88 months, and 60.9% were male children. The mean years of maternal education were 9 years, and nearly half of households (43.5%) were in the upper-income category. A majority of children (73.9%) experienced diarrheal duration lasting more than 72 h. Two days after recovery from diarrhea, the mean (SD) scores for cognitive, Language, and motor development were 97.28 (11.04), 97.87 (11.51), and 103.82 (11.50), respectively, and at the 90-day follow-up, the scores were 99.35 (10.12), 103.52 (12.01), and 105.36 (11.0), respectively.

**Table 1:** Characteristics of the children in the study (*N* = 138).

Characteristics	Mean (SD)
	*N* = 138
Child age (Bayley age in months)	10.84 (5.88)
Gender	
Male	84 (60.9%)
Female	54 (39.1%)
Maternal education (years)	9 (3)
Monthly income	
Lower income level (<12,500 BDT/Month)	28 (20.3%)
Middle income level (12500–21,500 BDT/Month)	50 (36.2%)
Upper income level (>21,500 BDT/Month)	60 (43.5%)
Crowding index	
≥3 persons/room	44 (31.9%)
<3 persons/room	94 (68.1%)
Diarrhea	
Diarrheal duration<72 h	36 (26.1%)
Diarrheal duration>72 h	102 (73.9%)
Handwashing during cooking	
Yes	51 (37%)
No	87 (63%)
Handwashing during feeding	
Yes	78 (56.5)
No	60 (43.5)
Fever	
Temp≥37.2 °C	31 (22.47)
Temp<37.2 °C	107 (77.53)
Nutritional status (Enrollment)	
Height-for-age Z score	−0.61 (1.02)
Weight-for-age Z score	−0.60 (1.13)
Weight-for-height Z score	−0.29 (1.15)
Nutritional status (90 days after diarrhea recovery)	
Height-for-age Z score (*n* = 138)	−0.79 (1.00)
Weight-for-age Z score (*n* = 129)	−0.40 (1.20)
Weight-for-height Z score (*n* = 128)	0.05 (1.18)
Difference of Height-for-age Z score	−0.18 (0.41)
Difference of Weight-for-age Z score	0.14 (0.50)
Difference of Weight-for-height Z score	0.27 (0.67)
Cognitive outcome (2 days after diarrhea recovery)	
Cognitive Composite Score	97.28 (11.04)
Language Composite Score	97.87 (11.51)
Motor Composite Score	103.82 (11.50)
Cognitive outcome (90 days after diarrhea recovery)	
Cognitive Composite Score	99.35 (10.12)
Language Composite Score	103.52 (12.01)
Motor Composite Score	105.36 (11.0)

Table 2 presents the associations of child, maternal socio-demographic characteristics, nutritional status, home environment, and morbidity with neurodevelopmental outcomes soon after recovery from diarrhea. Child age showed a significant positive correlation with Language (*ρ* = 0.23, *p* < 0.01) and a significant negative correlation with motor development (*ρ* = −0.26, *p* < 0.01). Additionally, girls had significantly higher Language scores than boys (*p* < 0.05). The quality of the home environment, measured using the HOME inventory, was positively associated with both cognitive (*ρ* = 0.25, *p* < 0.01) and Language development (*ρ* = 0.27, *p* < 0.01). On the other hand, a higher crowding index (≥3 persons/room) was significantly associated with a lower Language score (*p* < 0.05). Children who experienced diarrhea for more than 72 h had significantly lower mean language development score (96.72 ± 11.5) compared to those with a duration of less than 72 h (101.14 ± 10.9; *p* < 0.05). Among indicators of morbidity, only hospitalization was significantly associated with lower motor development (*p* < 0.05). No significant associations were observed between cognitive, language, or motor outcomes and caregiver education, household income, anthropometric indicators, handwashing practices, diarrheal severity, or fever status (all *p* > 0.05). Table 3 presents that several factors were associated with neurocognitive developmental outcomes at 90-day follow-up. Child age showed a significant negative correlation with cognitive (*ρ* = −0.19, *p* < 0.05) and motor (*ρ* = −0.21, *p* < 0.05) development. Additionally, female children had significantly higher mean language score (106.98 ± 10.86) compared to male children (101.30 ± 12.25); *p* < 0.01. Household income was positively correlated with the cognitive domain (*ρ* = 0.18, *p* < 0.05). Children whose mothers practiced handwashing during food preparation had significantly higher cognitive scores (101.96 ± 9.54) compared to those whose mothers did not (97.82 ± 10.19; *p* < 0.05). Similarly, handwashing during child feeding was associated with significantly higher language scores (105.47 ± 12.67 vs. 100.98 ± 10.67; *p* < 0.05). Height-for-age Z score showed a positive correlation with language development (*ρ* = 0.20; *p* < 0.05). The HOME inventory was also positively associated with language development (*ρ* = 0.26; *p* < 0.01). Multiple linear regression was conducted to see the association between diarrheal duration and neurodevelopmental outcomes after adjusting for age and sex of the children, household income, crowding index, height-for-age Z score, HOME, washing hands during food preparation and feeding children, hospitalization, and diarrheal duration (Table 4). Higher age was independently associated with lower scores in both the cognitive (*β* = −0.29; 95% CI: −0.87, −0.23; *p* = 0.001) and motor domains (*β* = −0.34; 95% CI: −0.98, −0.35; *p* < 0.001). Male sex was associated with higher language (*β* = 0.20; 95% CI: 0.72, 8.41; *p* = 0.020) and motor scores (*β* = 0.19; 95% CI: 0.78, 8.26; *p* = 0.018), but not cognitive (*p* = 0.077). Height-for-age Z score scores showed a positive but borderline association with language development (*β* = 0.16; 95% CI: −0.02, 3.73; *p* = 0.052). HOME was positively associated with cognitive (*β* = 0.35; 95% CI: 0.51, 1.80; *p* = 0.001) and motor development (*β* = 0.28; 95% CI: 0.32, 1.60; *p* = 0.004), but not language (*p* = 0.246). Hospitalization was independently associated with lower motor scores (*β* = −0.24; 95% CI: −11.35, −2.38; *p* = 0.003). Diarrheal duration >72 h was associated with lower language scores (*β* = −0.20; 95% CI: −9.52, −0.94; *p* = 0.017) but was not significantly associated with cognitive or motor domains.

**Table 2:** Association of child characteristics, maternal socio-demographic characteristics, nutritional status, and morbidity with neurocognitive development 2 days after recovery from diarrhea.

Variable	Cognitive		Language		Motor	
	*ρ*/Mean (SD)	*p* value	*ρ*/Mean (SD)	*p* value	*ρ*/Mean (SD)	*p* value
Child age^a^	−0.11	0.197	0.23	**<0.01**	−0.26	**<0.01**
Child sex^b^						
Male (*n* = 84)	96.01 (10.99)	0.092	96.27(11.53)	**<0.05**	102.44(11.01)	0.079
Female (*n* = 54)	99.26(10.92)		100.35(11.13)		105.96(12.0)	
Maternal education ^a^	−0.01	0.935	0.11	0.221	−0.03	0.732
Household Income ^a^	−0.04	0.687	0.10	0.244	−0.10	0.256
Crowding index ^b^						
≥3 persons/room (44)	96.14(10.05)	0.406	94.89(10.61)	**<0.05**	104.32(11.49)	0.728
<3 persons/room (94)	97.82(11.49)		99.27(11.70)		103.59(11.56)	
Handwashing during cooking ^b^						
Yes (*n* = 51)	97.45(12.38)	0.892	97.92(12.81)	0.968	105.76(11.68)	0.128
No (*n* = 87)	97.18(10.25)		97.84(10.75)		102.68(11.30)	
Handwashing during feeding ^b^						
Yes (*n* = 78)	97.76(9.86)	0.567	98.53(11.54)	0.447	104.24(11.38)	0.622
No (*n* = 60)	96.67(12.48)		97.02(11.51)		103.27(11.72)	
Height/Length-for-age Z score ^a^	0.09	0.279	0.14	0.10	0.03	0.703
Weight-for-age Z score ^a^	0.03	0.739	0.11	0.195	0.03	0.627
Weight-for-height/length Z score ^a^	−0.01	0.941	0.02	0.824	0.09	0.318
HOME total ^a^	0.25	**<0.01**	0.27	**<0.01**	0.10	0.230
Diarrheal duration ^b^						
<72 h (36)	97.50(11.1)	0.891	101.14(10.9)	**<0.05**	104.81(11.0)	0.551
>72 h (102)	97.21(11.04)		96.72(11.5)		103.47(11.7)	
Severity ^b^						
Mild (*n* = 81)	96.60(11.70)	0.392	99.01(11.73)	0.165	104.81(11.04)	0.625
Moderate to severe (*n* = 57)	98.25(10.07)		96.25(11.09)		103.47(11.69)	
Hospitalization ^b^						
Yes (*n* = 29)	96.55(9.74)	0.690	95.66(11.37)	0.245	**99.07(11.33)**	**<0.05**
No (*n* = 109)	97.48(11.40)		98.46(11.53)		105.08(11.26)	
Fever ^b^						
Temp≥37.2 °C (*n* = 31)	97.74(10.48)	0.794	99.61(11.48)	0.340	100.45(11.58)	0.064
Temp<37.2 °C (*n* = 107)	97.15(11.25)		97.36(11.52)		104.79(11.34)	

a Spearman rank correlation.

b Independent t-test; values are presented as Mean ± Standard Deviation (SD); HOME, Home Observations for Measurement of the Environment; *ρ*, Spearman correlation coefficient. The bold value is statistically significant.

**Table 3:** Association of child characteristics, maternal socio-demographic characteristics, nutritional status and morbidity with neurocognitive development at 90 days follow-up.

Variable	Cognitive		Language		Motor	
	*ρ*/Mean (SD)	*p* value	*ρ*/Mean (SD)	*p* value	*ρ*/Mean (SD)	*p* value
Child age ^a^	−0.19	**<0.05**	0.13	0.125	−0.21	**<0.05**
Child sex^b^						
Male (*n* = 84)	98.39(9.19)	0.168	101.30(12.25)	**<0.01**	105.08(10.58)	0.720
Female (*n* = 54)	100.83(11.36)		106.98(10.86)		105.78(11.87)	
Maternal education^a^	0.09	0.322	0.06	0.490	−0.06	0.493
Household Income^a^	0.18	**<0.05**	0.15	0.077	0.03	0.768
Crowding index ^b^						
≥3 persons/room (44)	98.07(8.97)	0.311	102.89(11.57)	0.672	107.48(10.51)	0.124
<3 persons/room (94)	99.95(10.61)		103.82(12.26)		104.36(11.23)	
Handwashing during cooking ^b^						
Yes (*n* = 51)	101.96(9.54)	**<0.05**	105.78(12.30)	0.090	106.41(10.89)	0.392
No (*n* = 87)	97.82(10.19)		102.70(11.71)		104.74(11.18)	
Handwashing during feeding ^b^						
Yes (*n* = 78)	99.94(10.15)	0.439	105.47(12.67)	**<0.05**	104.64(11.53)	0.389
No (*n* = 60)	98.58(10.13)		100.98(10.67)		106.28(10.45)	
Height/Length-for-age Z score (90 days) ^a^	0.07	0.425	0.20	**<0.05**	0.10	0.250
Weight-for-age Z score (90 days) ^a^	0.10	0.313	0.16	0.072	0.08	0.393
Weight-for-height/length Z score (90 days) ^a^	0.11	0.209	0.10	0.251	0.05	0.588
HOME total ^a^	0.05	0.597	0.26	**<0.01**	−0.07	0.391
Diarrheal duration						
<72 h (36)	98.89(9.2)	0.753	105.44(12.0)	0.265	106.56(10.8)	0.451
>72 h (102)	99.51(10.5)		102.84(11.9)		104.93(11.2)	
Severity ^b^						
Mild (*n* = 81)	99.63(9.80)	0.698	104.74(12.34)	0.156	106.22(10.27)	0.274
Moderate to severe (*n* = 57)	98.95(10.64)		101.79(11.41)		104.12(12.09)	
Hospitalization ^b^						
Yes (*n* = 29)	98.45(10.36)	0.692	102.34(10.98)	0.301	104.34(12.36)	0.595
No (*n* = 109)	99.59(10.10)		103.83(12.30)		105.62(10.74)	
Fever ^b^						
Temp≥37.2 °C (*n* = 31)	98.71(11.97)	0.637	101.55(11.20)	0.469	106.29(11.76)	0.332
Temp<37.2 °C (*n* = 107)	99.53(9.58)		104.09(12.22)		105.08(10.90)	

a Spearman rank correlation.

b Independent t-test; values are presented as Mean ± Standard Deviation (SD); HOME, Home Observations for Measurement of the Environment; *ρ*, Spearman correlation coefficient. The bold value is statistically significant.

**Table 4:** Multiple linear regression of cognitive, language & motor with diarrheal duration at soon after recovery adjusted for child age, sex, nutritional status, household income, crowding index, maintaining hygiene during cooking and feeding children, hospitalization and total home at soon after recovery of diarrhea.

Variables	Cognitive		Language		Motor	
	Regression coefficient (95%CI)	*p* value	Regression coefficient (95%CI)	*p* value	Regression coefficient (95%CI)	*p* value
Child age (months)	−0.29 (−0.87, −0.23)	**0.001**	0.14 (−0.07, 0.61)	0.114	−0.34 (−0.98, −0.35)	**<0.001**
Child sex (Male)	0.15 (−0.37, 7.10)	0.077	0.20 (0.72, 8.41)	**0.020**	0.19 (0.78, 8.26)	**0.018**
Household Income	0.07 (0.00, 0.00)	0.409	0.01(0.00, 0.00)	0.945	−0.08 (0.00, 0.00)	0.324
Crowing Index	−0.04 (−3.63, 5.36)	0.703	−0.08 (−6.50, 2.77)	0.427	0.14 (−1.14, 7.86)	0.142
Height for age Z score	0.03 (−1.50, 2.14)	0.726	0.16 (−0.02, 3.73)	**0.052**	−0.06 (−2.53, 1.11)	0.445
HOME total	0.35 (0.51, 1.80)	**0.001**	0.11 (−0.27, 1.05)	0.246	0.28 (0.32, 1.60)	**0.004**
Hand wash during Feeding	−0.02 (−4.91, 4.06)	0.850	0.09(−2.57, 6.68)	0.381	−0.08(−6.29, 2.69)	0.429
Hand wash during cooking	−0.05 (−0.53, 3.40)	0.638	−0.07 (−6.34, 2.86)	0.456	0.09(−2.28, 6.66)	0.335
Hospitalization	−0.05(−5.80, 3.15)	0.559	−0.09 (−7.08, 2.15)	0.293	−0.24(−11.35, −2.38)	**0.003**
Diarrheal duration>72	−0.05 (−5.47, 2.86)	0.536	−0.20 (−9.52, −0.94)	**0.017**	−0.09 (−6.39, 1.95)	0.294

The bold value is statistically significant.

Table 5 presents the results of multiple linear regression models at 90 days follow-up, adjusting for children’s age, sex, household income, crowding index, height-for-age Z score, HOME, washing hands during food preparation and feeding children, hospitalization, and diarrheal duration. Child age was significantly associated with lower cognitive scores (*β* = −0.20; 95% CI: −0.64, −0.03; *p* = 0.030), but not with language or motor outcomes. Male sex was significantly associated with higher language scores (*β* = 0.22; 95% CI: 1.49, 9.34; *p* = 0.007), but not with cognitive or motor scores. Handwashing during cooking was positively associated with cognitive scores (*β* = 0.23; 95% CI: 0.73, 8.73; *p* = 0.021). HOME scores showed a positive but borderline association with language development (*β* = 0.18; 95% CI: −0.01, 1.31; *p* = 0.054). Handwashing during feeding also approached significance in relation to motor development (*β* = −0.19; 95% CI: −8.60, 0.02; *p* = 0.051).

**Table 5:** Multiple linear regression of cognitive, language & motor with diarrheal duration at 90 days follow-up adjusted for child age, sex, nutritional status, household income, crowding index, maintaining hygiene during cooking and feeding children, hospitalization, total home and initial neurocognitive development.

Variables	Cognitive		Language		Motor	
	Regression coefficient (95%CI)	*p* value	Regression coefficient (95%CI)	*p* value	Regression coefficient (95%CI)	*p* value
Child age (months)	−0.20 (−0.64, −0.03)	**0.030**	−0.02 (−0.37, 0.31)	0.853	−0.14(−0.60, 0.07)	0.120
Child sex (Male)	0.09 (−1.45, 5.31)	0.260	0.22 (1.49, 9.34)	**0.007**	0.02 (−3.14, 4.17)	0.780
Household Income (monthly)	0.15 (0.00, 0.00)	0.077	0.10 (0.00, 0.00)	0.213	0.11 (0.00, 0.00)	0.177
Crowding Index	−0.03(−4.59, 3.47)	0.783	0.15(−0.83, 8.44)	0.107	0.14(−0.96, 7.74)	0.125
Height-for-age Z score	0.03 (−1.37, 1.90)	0.750	0.13 (−0.44, 3.36)	0.130	0.12 (−0.47, 3.02)	0.152
HOME total	0.01 (−0.57, 0.64)	0.908	0.18 (−0.01, 1.31)	**0.054**	0.01 (−0.59, 0.68)	0.887
Handwashing during feeding	−0.12 (−6.52, 1.51)	0.220	0.04 (−3.56, 5.70)	0.648	−0.19 (−8.60, 0.02)	**0.051**
Handwashing during cooking	0.23 (0.73, 8.73)	**0.021**	0.09 (−2.31, 6.89)	0.326	0.10 (−2.12, 6.47)	0.318
Hospitalization	−0.02(−4.43, 3.60)	0.838	−0.03(−5.62, 3.63)	0.671	0.03(−3.59, 5.30)	0.704
Diarrheal duration>72	0.04 (−2.82, 4.65)	0.628	−0.06 (−6.03, 2.72)	0.454	−0.04 (−5.01, 3.00)	0.621

The bold value is statistically significant.

## Discussion

This study primarily investigated the neurocognitive outcomes of children under 2 years of age who had recovered from watery diarrhea, assessed at 2- and 90-days post-illness, in rural Mirzapur, Bangladesh. Findings show that cognitive, language, and motor scores, assessed 2 days after recovery, gradually improved over the 90-day follow-up period. This finding suggests a temporary decline in developmental performance following acute watery diarrhea, with improvement occurring over time. The improvement was more in the language domain (around 5 points) compared to the cognitive and motor domains (around 2 points). Furthermore, child age, sex, growth, home stimulation, handwashing behavior, duration of diarrheal illness, and hospital admission were significantly and independently associated with different domains of development.

Prolonged diarrheal illnesses in children below 2 years of life can cause developmental disadvantages through multiple, overlapping pathways. One possible mechanism is gut-brain axis disruption resulting in recurrent enteric infections and environmental enteric dysfunction (EED), impairing nutrient absorption and triggering inflammation during critical growth periods (29, 30). The other possible explanation may be diarrhea-related growth faltering or malnutrition, risk, both of which are known risks for delayed cognitive and language development (13). In our study, young children with watery diarrhea lasting longer than 72 h scored significantly lower in language development than those with a shorter duration of illness (less than 72 h) 2 days after recovery (96.72 ± 11.5 vs. 101.14 ± 10.9; *p* < 0.05). However, this difference was no longer significant at the 90-day follow-up (105.44 ± 12.0 vs. 102.84 ± 11.9, *p* = 0.265), both in the unadjusted and in the adjusted model. Interestingly, these scores remained within the normal range (100 ± 15) based on U. S. norms at both assessment time points, possibly due to a single episode of watery diarrhea with almost no recurrence in 90 days of follow-up. The findings suggest that a single medium-duration episode of such illness may exert a transient and reversible adverse effect, especially in language development. This temporary reduction is not unexpected, as children recovering from acute illnesses often experience fatigue or lethargy, leading to decreased engagement in both verbal and non-verbal (gesture) communication. The quick catch-up of initial delay in the language domain in a 90-day follow-up can also happen due to the contribution of other factors like improved nutrition, hygiene practices, and home care (31). No significant differences were observed in other assessed neurocognitive domains, such as cognitive and motor functions, between children who experienced shorter versus longer durations of watery diarrheal illness, both shortly after recovery and at the 90-day follow-up.

We found very few studies that looked for the association of diarrhea with development among young children and reported detrimental neurodevelopmental outcomes (11, 29, 32, 33). The South Indian study (33) explored the association of the total number of diarrheal days over 6 months in young children and found that prolonged duration of diarrheal illness was specifically associated with reduced fine motor and problem-solving abilities of the children. Another study in Bangladesh (32) evaluated 211 hospital-admitted young diarrheal children with high serum sodium levels (>159 mmol/L) who scored low in cognitive, language, and motor development after recovery, and that did not improve over time for language or motor domains at 12 months follow-up. Likewise, the long-term community-based cohort study in Brazil (11) reported sustained adverse developmental outcomes among 131 children at 8 years of age who had experienced multiple bouts of diarrheal episodes during the first 2 years of life. The community had a high burden of diarrhea, with a median of 5.0 episodes with a mean duration of 3.3 days over 24 months, and the children with higher episodes of diarrhea scored significantly low in all the measured domains of development (verbal fluency, coding, and non-verbal intelligence). Earlier reports of this surveillance also showed a significant association of diarrheal episodes with nonverbal intelligence, attention, processing speed, memory tasks, visual-motor coordination, and school functioning at school age (6). A subset of these children with persistent diarrhea (duration >14 days) specifically scored low in the *Coding* and *Digit Span* at 6 to 10 years of age. Although these studies are not fully comparable to our study because of the severity of illnesses, timing of assessments and follow-up, measurement of domains, use of assessment tools, and study contexts, their consistent findings suggest that prolonged duration and severity of diarrhea in early childhood may have lasting adverse effects on development and learning outcomes at school age. So, from our current findings, it remains difficult to determine whether any detrimental effects on developmental domains emerge later in childhood.

Among the other covariates that showed independent associations with developmental scores, child age showed a significant but inverse association with cognitive and motor ability, but not with language performance, and persisted for cognitive outcomes at the 90-day follow-up. This trend of a sustained decline in cognitive scores with increasing age may suggest greater vulnerability of children to neurodevelopmental impacts when exposed to morbidities such as diarrheal illness. Previous studies also showed a similar decline of cognitive scores over time with increasing age due to the cumulative effect of adversity, e.g., poverty, malnutrition, lack of stimulation, etc. (34, 35). Inverse association of age with developmental scores possibly influenced by the use of assessment tools standardized on Western populations, although culturally and contextually adapted for our settings (36, 37). However, to address these issues, we adjusted for all the potential confounders in the analysis, e.g., nutritional status, household income, crowding index, maintaining hygiene during cooking and feeding children, hospitalization, and home stimulation.

Overall, our study population was not malnourished, and height for age z score showed a positive association with cognition soon after recovery from diarrhea that approached significance [regression coefficient (95% CI): 0.16 (−0.02, 3.73), p 0.052]. Our study also revealed that male children performed significantly better in language and motor domains compared to females soon after recovery, but at 90 days of follow-up, this association sustained for language outcomes only. A systematic review and meta-analysis (ages 3–6) similarly showed that boys outperform girls in object control skills (throwing, catching) with a moderate effect size, and this gender gap widens with age (38). From ages 5 to 16, boys have been found to outperform girls in areas such as oral language comprehension, spatial reasoning, and visual perception, although girls maintain an advantage in tactile tasks (39, 40). Collectively, these findings suggest that boys may engage motor and language pathways differently during the acute and early recovery phases of illness. Consistent with previous literature (41, 42), in these young children, higher HOME scores, indicating the amount of stimulation and care at home, showed a significant positive association specifically with cognitive and motor development soon after recovery from diarrhea and approached significance with language development after 90 days of assessment. Similarly, handwashing during feeding and cooking showed a significant positive association with development at 90-day follow-up. This indicates that both a stimulating home environment and handwashing practices are protective for cognitive development. The severity of watery diarrhea in our study, captured by hospitalization during diarrhea, was also associated with lower motor outcomes shortly after recovery. Children hospitalized for severe watery diarrhea likely experience more pronounced nutrient depletion, electrolyte imbalance, and systemic inflammation (43, 44). Moreover, hospitalization typically restricts children’s physical mobility and reduces exposure to enriching environments. These conditions can contribute to temporary setbacks in motor skill recovery. Although not explicitly studied in this context, broader literature on early development supports how low stimulation delays motor recovery (33).

We have a few limitations in our study. First, the relatively small sample size may have limited statistical power to detect subtle associations, particularly in adjusted analyses. Secondly, although the updated version of the Bayley-4 scale was culturally adapted, it has not been standardized for Bangladeshi children, which could affect the accuracy of developmental scores. Lastly, confounders, such as micronutrient status and gut microbiota composition, could also influence neurocognitive outcomes but were not assessed.

This study is one of the few to prospectively investigate the relationship between watery diarrhea and neurocognitive development among children under 2 years of age in a rural low-resource setting. We used validated and widely applied tools, including the updated version of Bayley-4 and the HOME inventory, to capture multiple dimensions of child development and the quality of the home environment. Although the Bayley-4 is not standardized for Bangladeshi children, measurement error was minimized by testing only healthy, alert, and comfortable participants. The tool was culturally adapted and has previously shown sensitivity to intervention effects in Bangladesh. Assessors completed 1 month of intensive training and were certified upon achieving >95% inter-rater agreement with trainers. Repeated assessments at two time points enabled the examination of both immediate and short-term effects of diarrheal illness on developmental outcomes. Additionally, the study controlled for a comprehensive range of sociodemographic and health-related confounders, including anthropometry, thereby strengthening the validity and robustness of the findings.

## Conclusion

This study demonstrates that watery diarrhea in early childhood is associated with short-term impairments in language and motor development, particularly among children with prolonged diarrhea episodes, hospitalization, or who are stunted. Although many of these effects recovered within 90 days, boys consistently performed better than the girls in language, while older age consistently predicted poorer cognitive outcomes, highlighting a window of vulnerability during infancy and toddlerhood. At the same time, enriched home stimulation and household hygiene practices were protective, supporting better developmental recovery. These findings underscore the dual importance of clinical management of diarrhea and interventions that strengthen the caregiving environment. Further research is needed to explore whether watery diarrhea has long-term effects on a child’s cognitive, language, and motor development, including the underlying mechanisms through which these effects occur, and to examine these associations across diverse populations in order to inform the design of effective interventions in low-resource settings.

## Data Availability

The raw data supporting the conclusions of this article will be made available by the authors, without undue reservation.
